# Assessing spatiotemporal variation in abundance: A flexible framework accounting for sampling bias with an application to common pochard (*Aythya ferina*)

**DOI:** 10.1002/ece3.8835

**Published:** 2022-04-20

**Authors:** Benjamin Folliot, Alain Caizergues, Adrien Tableau, Guillaume Souchay, Matthieu Guillemain, Jocelyn Champagnon, Clément Calenge

**Affiliations:** ^1^ Office Français de la Biodiversité Direction de la Recherche et de l’Appui Scientifique Nantes France; ^2^ DYNECO ‐ Laboratoire d’écologie benthique Ifremer, Centre de Bretagne ZI de la Pointe du Diable Plouzané France; ^3^ Office Français de la Biodiversité Direction de la Recherche et de l’Appui Scientifique Arles France; ^4^ Tour du Valat Research Institute for the Conservation of Mediterranean Wetlands Arles France; ^5^ Office Français de la Biodiversité Direction Surveillance, Evaluation, Données – Unité données et appui méthodologique Saint Benoist France

**Keywords:** common pochard, ducks, hierarchical modeling, population trends, sampling bias, spatial autocorrelation

## Abstract

Assessing trends in the relative abundance of populations is a key yet complex issue for management and conservation. This is a major aim of many large‐scale censusing schemes such as the International Waterbird Count (IWC). However, owing to the lack of sampling strategy and standardization, such schemes likely suffer from biases due to spatial heterogeneity in sampling effort. Despite huge improvements of the statistical tools that allow tackling these statistical issues (e.g., GLMM, Bayesian inference), many conservationists still prefer to rely on stand‐alone turn‐key statistical tools, often violating the prerequisites put forward by the developers of these tools. Here, we propose a straightforward and flexible approach to tackle the typical statistical issues one can encounter when analyzing count data of monitoring schemes such as the IWC. We rely on IWC counts of the declining common pochard populations of the Northwest European flyway as a case study (period 2002–2012). To standardize the size of sampling units and mitigate spatial autocorrelation, we grouped sampling sites using a 75 × 75 km grid cells overlaid over the flyway of interest. Then, we used a hierarchical modeling approach, assessing population trends with random effects at two spatial scales (grid cells, and sites within grid cells) in order to derive spatialized values and to compute the average population trend at the whole flyway scale. Our approach allowed to tackle many statistical issues inherent to this type of analysis but often neglected, including spatial autocorrelation. Concerning the case study, our main findings are that: (1) the northwestern population of common pochards experienced a steep decline (4.9% per year over the 2002–2012 period); (2) the decline was more pronounced at high than low latitude (11.6% and 0.5% per year at 60° and 46° of latitude, respectively); and, (3) the decline was independent of the initial number of individuals in a given site (random across sites). Beyond the case study of the common pochard, our study provides a conceptual statistical framework for estimating and assessing potential drivers of population trends at various spatial scales.

## Graphical abstract

In this paper, we focused on analysing winter count data for common Pochard to estimate the trend in population size between the years 2002 and 2012. These data are routinely analysed to inform the IUCN conservation status of species. However, we highlight the existence of statistical biases that need to be taken into account to make the analyses more robust. For the species studied, our analysis shows a significant decrease in population size over the period studied, justifying the vulnerable nature of its conservation status. Moreover, the analysis tool we developed allowed us to highlight a more profound decrease to the north of the flyway than to the south, evidence that could not be highlighted with the classic tools usually used. Hence, given the growing interest by conservationists, we developed an R package to allow anyone using this model and reproducing the analysis done.

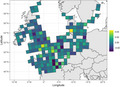

## 1 INTRODUCTION

Wildlife populations are often distributed over huge areas, especially in migratory species (e.g., common pochards occupy continental flyways, Folliot et al., 2018), making difficult the implementation of reliable strategies for assessing their abundance and temporal trends (Jarman et al., 1996). Counting migratory birds, whose distribution often encompasses whole continents, requires the coordination of censuses and surveys over numerous entities (states, provinces, and countries) not necessarily sharing the same management priorities or conservation status for the same species. Nevertheless, the monitoring of migratory birds has a long tradition of coordinated “transnational” population censuses (Amano et al., 2018; Gregory et al., 2007; Lehikoinen et al., 2013; Sayoud et al., 2016). For example, Wetlands International coordinates the International Waterbird Census (IWC) over more than 100 countries around the world since 1967 (Amano et al., 2018). In the Palearctic region, the management of Anatidae species is conducted at the flyway level based on the IWC (censuses performed around January 15, Elmberg et al., 2006). These counts, together with censuses performed during the breeding season, serve to establish the International Union for Conservation of Nature (IUCN)’s red lists (IUCN, 2020), and implement management actions. In Western Europe, the red list status of harvested species helps implementing hunting policies by member countries of such agreements as the African–Eurasian Waterbird Agreement (AEWA).

Large‐scale censuses and surveys also serve scientific purposes. The internationally coordinated censuses, such as IWC, the Mid‐Winter Survey, or the Christmas Bird Count (MWS or CBC respectively, its North American counterparts) and the Pan‐European Common Bird Monitoring Scheme (PECBMS), have recently been used to assess the efficiency of conservation policies (Amano et al., 2018; Gaget et al., 2020; Jørgensen et al., 2016), birds’ response to climate change (Lehikoinen et al., 2013; Meehan et al., 2021; Pavón‐Jordán et al., 2019), or both (Gaget et al., 2018).

Unfortunately, international bird censuses, as those carried out in the Palearctic area, potentially suffer weaknesses that should be addressed adequately in the analyses. The methods required to model the population size from the censuses data depend on the type of collected data. In most large‐scale monitoring, models used to infer population relative abundance rely on the assumption that the population abundance estimated in a site can be used to infer the abundance in the surrounding area. For example, most models of the CBC data suppose that the counted numbers of birds in all sites in a given region (“stratum”) are similar enough to allow the inference of an average relative abundance for the region (Soykan et al., 2016). Models of North American Breeding Bird Survey (BBS) rely on a similar assumption, with all the routes belonging to a given region used to estimate the average relative abundance of the birds in this region (Link & Sauer, 1998; Sauer & Link, 2011). This similarity of bird abundances on routes close to each other is often more explicitly accounted for using spatial models (Bled et al., 2013; Thogmartin et al., 2004).

However, considering waterbirds, we cannot expect that bird abundances are similar in close locations: the number of ducks is likely to be very different on a small pond and on the neighboring lake. Therefore, a model of censuses cannot suppose that the abundance of water birds on a given water point is similar to the abundance on water points of the surrounding region, precluding the use of classical regression models such as those developed for BBS or CBC. On the other hand, even if adjacent entities are not characterized by similar bird abundances, they may potentially be sharing the same demographic characteristics, in particular population trends (Knape & de Valpine, 2012). This suggests that a modeling approach allowing to infer average population trends in regions without attempting to estimate the population abundance might be useful for such species.

When the IWC scheme was implemented, each country was asked to maximize the inferential potential of their monitoring efforts by focusing on more abundant sites to allow for more accurate estimation of populations trends, but no other sampling strategy was proposed to help achieving these goals (Delany, 2010). Therefore, censuses schemes were initially designed to be easily implemented at large scale to involve as many countries/territories as possible and almost entirely lacked a real sampling strategy. As a result, the managers in charge of the counts tended to prefer biodiversity hotspots for monitoring. Sampled sites may differ in size, densities of individuals, observation pressure, skill of observers, census methods, and so on, yet these differences are most of the time completely ignored in the analyses. As a consequence, overdispersion often “plague” the datasets and may seriously affect both estimate values and variable selection. Indeed, unaccounted overdispersion leads to an overestimated precision of estimates, and thereby to an inflated type I error rate (e.g., a random between‐year variation in the estimated population size considered as a significant change in actual population size, or an environmental variable wrongly considered as having a significant effect on the population size; Cameron & Trivedi, 1998, chap. 3). For this reason, most authors include overdispersion residuals in their models of census data (Link & Sauer, 1998; Sauer & Link, 2011) or use statistical distributions other than Poisson (e.g., quasi‐Poisson approaches, Pannekoek & van Strien, 2001). Finally, some data may be missing (Komdeur et al., 1992) leading to some difficulties in the inference of the population size and trends.

In summary, the lack of sampling strategy and standardization over time and space, together with the other problems mentioned above, may lead to important biases that should be considered in the analyses of wildlife census data. To date, the assessment of population trends often relies on a “standalone turn‐key solution” (usually TRIM software, Lehikoinen et al., 2013; Musil et al., 2011; Musilová et al., 2018), which has proven invaluable but suffers from drawbacks (Amano et al., 2018 or Meehan et al., 2021). TRIM tackles overdispersion but does not allow accounting for the problem of spatial autocorrelation in the population trends. However, most TRIM users, so far, simply ignored spatial autocorrelation together with the possibility to tackle overdispersion, meaning that in numerous instances the models did not fit the data. Trying to circumvent this last problem, some authors opted for a “last resort” solution consisting in performing analyses on log‐transformed counts in a normal distribution framework (Pavón‐Jordán et al., 2020), a strategy generally resulting in biased estimates when the count data include many zeroes (O’Hara & Kotze, 2010).

Here, we present a flexible statistical approach aimed at providing unbiased estimates of temporal trends in numbers derived from censuses collected in different countries and hence, potentially suffering the same biases as discussed above. We use the IWC data of common pochard (*Aythya ferina* – hereafter pochard) in the Northwestern European flyway as a case study. This diving duck is considered to have experienced a sharp decline over most of its range including the focal flyway (mean annual declines of −5.97% and −2.16% in the Northwestern and Central European Flyways, respectively, as estimated with TRIM, 2003–2012, Nagy et al., 2014). Such unfavorable status has caused the species to be upgraded from Least Concern (LC) to Vulnerable (VU) on the European and global IUCN Red Lists in 2015 (BirdLife International, 2015), and demographic studies to be initiated (Caizergues et al., 2016; Folliot et al., 2017, 2018, 2020; Gourlay‐Larour et al., 2012, 2013, 2014; Keller, 2009).

The aims of the study were as follows: (1) to exemplify how biased a typical IWC dataset can be, (2) to develop a hierarchical modeling framework that adequately considers overdispersion and spatial autocorrelation in relative abundance trends, and (3) to assess within this framework the trends of pochard populations in Northwestern Europe, taking into account possible differences between the different parts of the wintering range in this area. We selected the IWC’s pochard dataset as a case study both because it included the typical biases of interest mentioned above, and because these biases potentially plague many waterbird count datasets including those of IWC.

## 2 MATERIALS AND METHODS

**FIGURE 1:**
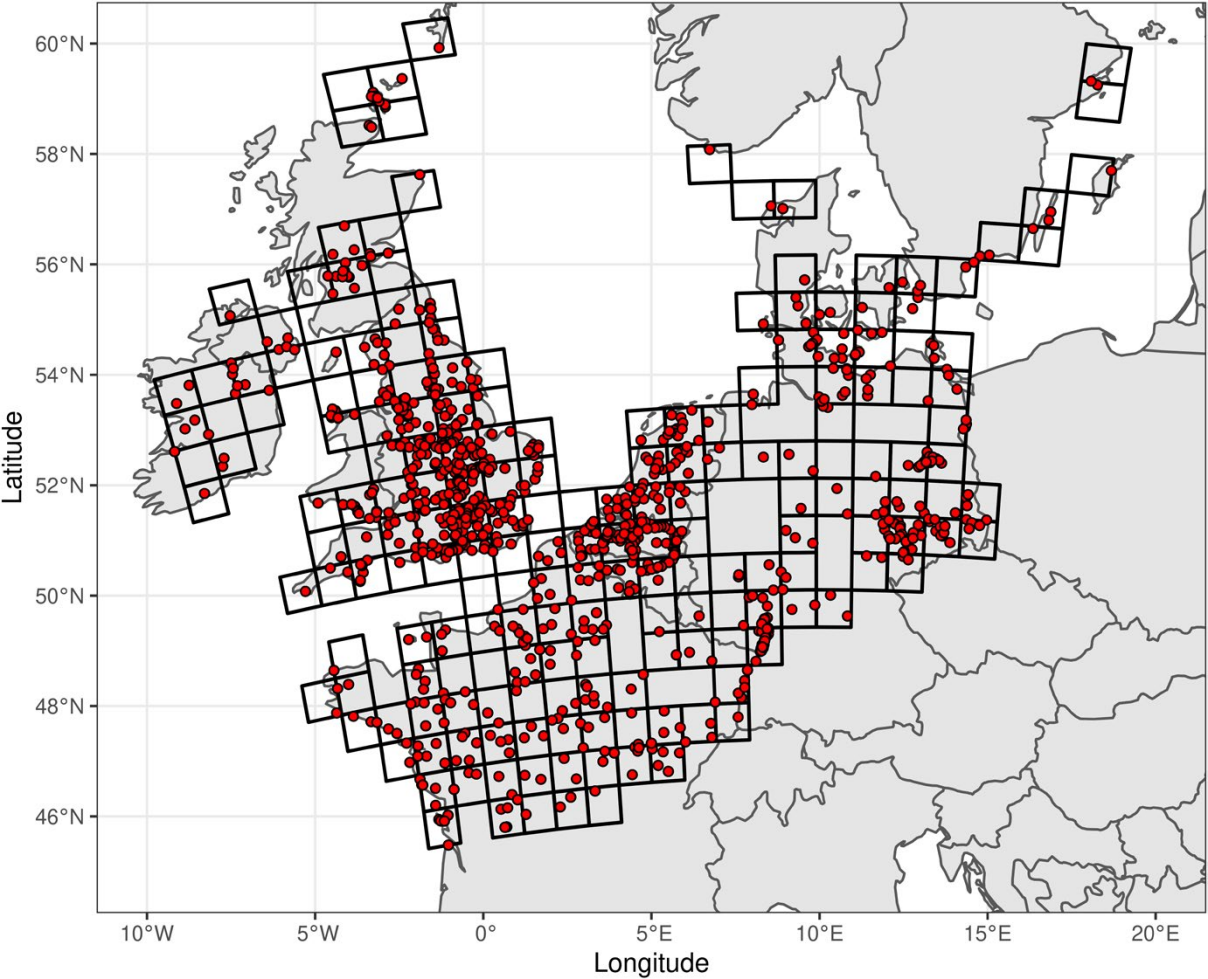
Spatial distribution of the 981 sites surveyed at least 7 years between 2002 and 2012 (red circles) located in the northwestern flyway with the juxtaposition of 193 grid cells (75 × 75 km) when at least one site surveyed is present for the monitoring of the common pochard (*Aythya ferina*)

The pochard is a hunted diving duck whose distribution encompasses the Palearctic region (i.e., Western Eurasia and North Africa), mostly above 40° of latitude (Folliot et al., 2018; Fox et al., 2016). We focused on pochard populations of the Northwestern European flyway. For the sake of simplicity (to avoid tackling, e.g., threshold effects often characterizing long‐term count time series) and comparison with previous estimates, we focused on the January IWC counts of the 2002–2012 decade. During this period, the dataset covered 3273 sites counted at least once. Since it is difficult to assess the long‐term trend of population size on a site using only a few counts, we focused our modeling approach on those sites counted at least 60% of the time (7 times) during the study period, representing a total of 981 sites (Figure 1).

### 2.1 Assessing the nature of the problem

Before designing the statistical approach for the assessment of the population trend, we first fitted a preliminary simple Poisson generalized linear mixed model of the number of pochards on a site during a given year as a linear function of the year. This model included a site‐specific intercept and a random effect of the site on the slope of the year. Indeed, while the slopes of N sites can be considered as N noisy measurements of an average trend, the intercepts (average number of individuals) may display consistent differences between sites due to, for example, differences in site size area. This model also included overdispersion residuals, and was fitted using the package TMB for the R software (a package that accommodate both large volume of data and large number of parameters, Kristensen et al., 2016). This analysis revealed that this period was characterized by a steep linear decline in the number of individuals counted (mean slope equal to −0.04, SE = 0.005; although this SE probably overestimates the precision of the slope, as it does not account for the spatial autocorrelation in the trends, see below). We assessed the spatial autocorrelation of the estimates of regression slopes estimated for each site with a Moran test (Cliff & Ord, 1981) based on a relative neighborhood graph (Toussaint, 1980, spdep package – Bivand et al., 2015). Moran's test detected highly significant but weakly positive spatial autocorrelation (Moran statistic *I* = 0.08, *p* = .006), meaning that neighboring sites tended to display similar temporal trends. A map showing the distribution of the sampled sites suggests that this spatial autocorrelation was due to the spatial heterogeneity of sampling effort, with some areas being oversampled (Figure 1). In some areas, the distance between neighboring sampling sites was in some cases lower than 100 m, leading to a high probability that the same groups of birds were counted on neighboring sites. Not accounting for this unequal sampling intensity would lead to a disproportionate weight of oversampled areas in the analysis, leading to overall trend estimates strongly affected by the local trends observed in these areas.

### 2.2 Using a grid to ensure the standardization of sampling units

We designed a hierarchical modeling approach to account for spatial autocorrelation and non‐random site selection (sampling heterogeneity). To ensure the reproducibility of the analyses carried out in this study, we included all the code and data used for this modeling approach in the R package pochardTrend (Digital Object Identifier: https://doi.org/10.5281/zenodo.5710550) on GitHub at the following URL: https://github.com/ClementCalenge/pochardTrend. The reader can install this package in R with the package devtools (Wickham et al., 2021), using the function devtools::install_github ("ClementCalenge/pochardTrend", ref="main"). The pochardTrend package includes a vignette describing how the user can easily reproduce the model fit (available with the command vignette ("pochardTrend"), once the package has been installed). We wrote this vignette to provide the user with the essential information on the model. This vignette also describes the model checks and residual analysis carried out for the obtained model (see below).

To account for spatial sampling heterogeneity, we “standardized” the size of sampling areas using a grid made of 193 (75 × 75 km) grid cells encompassing the flyway of interest (Figure 1). Each grid cell included at least 1 of the 981 sites retained in the analyses (see above). We opted for such a spatial resolution because it offered the best compromise between a low resolution and an overly high one that would have included non‐sampled territories (grid cells). Therefore, each “sampling unit” covered a standard area and all the sampling units had the same weight in the analysis. Shifting from a set of sampled points with an irregular geographical repartition to a lattice process is a common strategy to standardize the area sampling unit and give them the same weight in the analysis (Bled et al., 2013).

### 2.3 Structure of the hierarchical model

A major aim of our study was to develop a hierarchical model for estimating population trends from IWC‐like data. We therefore fitted a model of the form:

(1) $$N_{\mathrm{it}}∼\text{Poisson}\left(\lambda_{\mathrm{it}}\right)$$

Where *N_it_* is the number of individuals detected in site *i* during year *t*. We described these numbers using a Poisson distribution with mean $\lambda_{\mathrm{it}}$, supposing the following model for this parameter:

(2) $$\log\left(\lambda_{\mathrm{it}}\right)=\alpha_{i}+\left(\beta_{0}+d_{i}\right)\cdot t+e_{\mathrm{it}}$$

$e_{\mathrm{it}}$ is a Gaussian residual characterizing site *i* and year *t* (with mean 0 and standard deviation $\sigma_{e}$) included in the model to account for overdispersion; $\alpha_{i}$ is the intercept of the model for site *i* (Pannekoek & Van Strien, 2001; Sauer & Link, 2011), the slope of the year *t* is the sum of a fixed slope $\beta_{0},$ and a random site effect *d_i_* modeled using a Gaussian distribution with a mean $b_{q\left(i\right)}$ characterizing grid cell *q(i)* containing the site *i* and a standard deviation equal to $\sigma_{d}$:

(3) $$d_{i}∼\text{Normal}\left(b_{q\left(i\right)},\sigma_{d}\right)$$

Note that if we calculate the sum $\beta_{0}+b_{q\left(i\right)}$, the resulting value is the slope of the relationship between the log‐mean number of animals in grid cell *q(i)* and the year. The parameter *b_q(i)_* is itself modeled by a Gaussian distribution:

(4) $$b_{q\left(i\right)}∼\text{Normal}\left(\gamma \cdot L_{q\left(i\right)},\sigma_{b}\right)$$

where γ is the slope of the latitude *L_q(i)_* of the grid cell *q(i)* containing the site *i* and $\sigma_{b}$ is the standard deviation of this Gaussian distribution.

Here, we modeled the trend as a function of the latitude only, but it would in theory be possible to model the slope as a function of other environmental variables (e.g., land cover change). We focused on the latitude to assess possible differences in trends over the species’ range on a North–South axis due to migratory short stopping (shortening of migration distance leading to increasing numbers at high latitudes and decreasing numbers at low latitudes) in response to increasing winter temperatures (Elmberg et al., 2014; Tománková et al., 2013).

To summarize, this hierarchical approach estimated (1) the population trend $\beta_{0}+b_{q\left(i\right)}$ in each grid cell and (2) the average population trend $\beta_{0}$ over the whole flyway of interest (here the Northwestern European flyway).

To give more meaningful measure of the trends, we estimated the median percentage r of decrease in the population at a given latitude *L* over a duration *D* by calculating:

(5) $$r=100\times \left[1‐\exp\left(D\times \left(\beta_{0}+\gamma \cdot L\right)\right)\right]$$

We calculated this percentage at latitudes 60° and 46°, for durations of 1 year and 10 years.

In order to avoid convergence issues caused by correlations between the intercept and the slope, we centered year (*t*) at the year 2007 (middle of the study period, following the recommendation of Pinheiro and Bates (2000) to center explanatory variables before the fit). The model ran on R software (R Core Team, 2013) using the TMB package (Kristensen et al., 2016). Finally, we tested the existence of a remaining spatial autocorrelation in the random effects $d_{i}$ and $b_{q\left(i\right)}$ with a Moran test.

## 3 RESULTS

The strategy of standardization of sampling units through the use of grid cells proved effective to tackle the problem of spatial autocorrelation at both the grid cell and site levels (grid cell level: Moran test *I* = 0.060, *p*‐value = .14; site level: Moran test *I* = 0.012, *p*‐value = .33). Moreover, the random estimates of slopes for both the site and grid cell did not show any departure from normality, suggesting that the model correctly fitted the data. Finally, the plot of the residuals of the model against year did not exhibit any particular pattern, meaning that the observed trend did not depart from log‐linearity.

**FIGURE 2:**
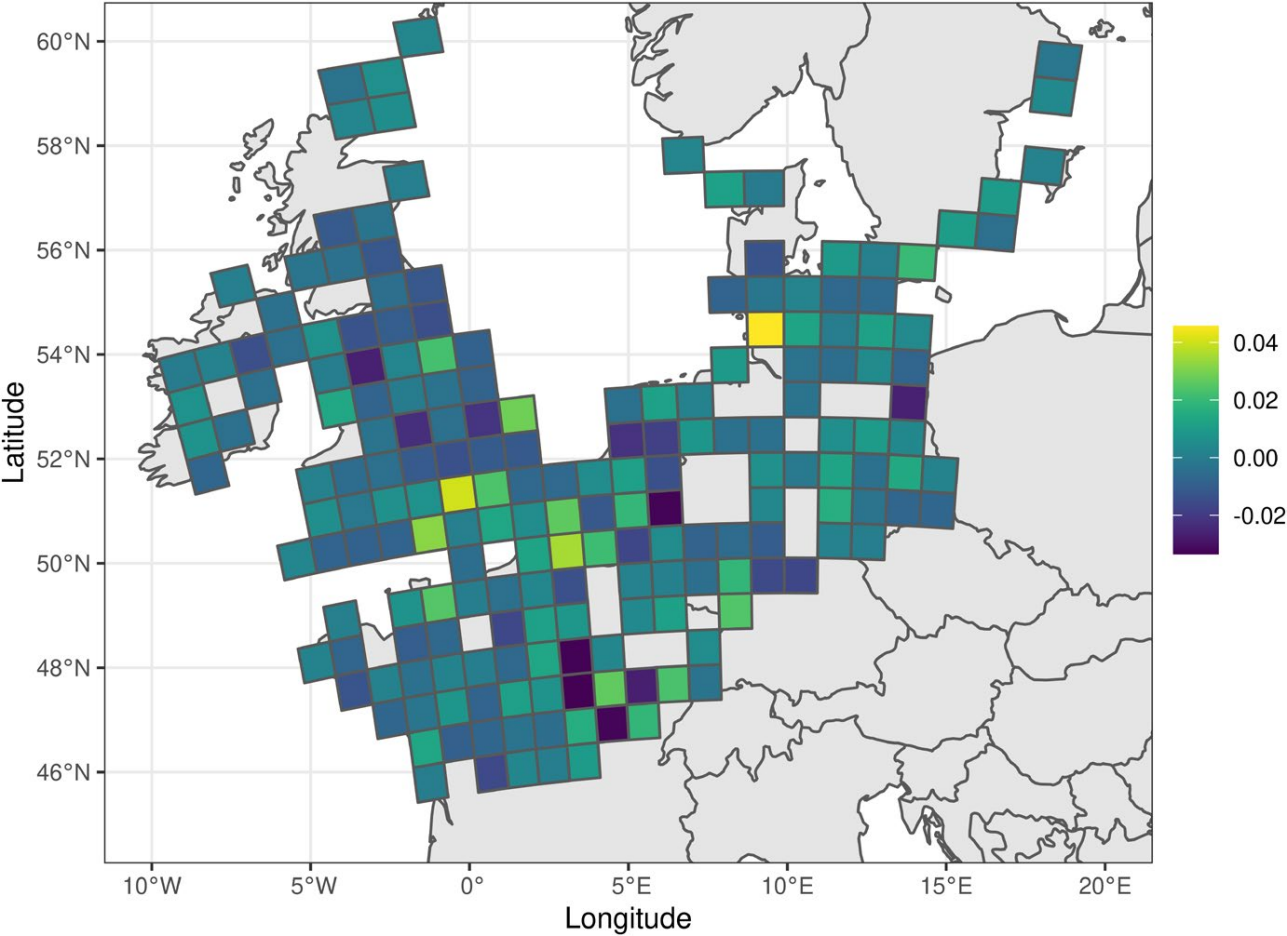
Spatial distribution of the random effects of population trends of common pochards (average changes in the logarithm of numbers of individuals per year) at the grid cell scale

We identified a significant negative effect of the latitude of a grid cell on the population trend (Table 1). The decline in the number of pochards estimated using our hierarchical model (Equation 5) was greater at northern than at southern latitudes. Based on our model, we estimated a significant decrease in population size equal to 74% over the entire period for latitudes of 60° (SE = 6%; corresponding to a mean decrease of 11.6% per year, SE = 1.8% – calculation carried out within the developed R package vignette). In contrast, the decline was not significant in grid cells at 46° of latitude (mean decrease of 5.7%, SE =14%; corresponding to a mean decrease of 0.54% per year, SE = 1.4%, Figure 2).

**TABLE 1:** Parameter estimates and their standard errors derived from the GLMM model assessing the spatio‐temporal variations in numbers of pochards in the Northwestern European flyway

Parameter	Estimation	SE
Year ($\beta_{0}$)	0.38	0.12
Latitude (γ)	−0.008	0.002
*σ_b_*	0.033	0.001
*σ_d_*	0.073	0.008
*σ_e_*	1.18	0.01

Note

Year is for the global slope parameter $\beta_{0}$ associated with the year in the model; Latitude is the slope coefficient γ of latitude on the random effect of the grid cells. The parameters *σ_e_*, *σ_d_*, and *σ_b_* are the standard deviations of the overdispersion residuals, the site random effects, and the random effects of the grid cells, respectively.

**FIGURE 3:**
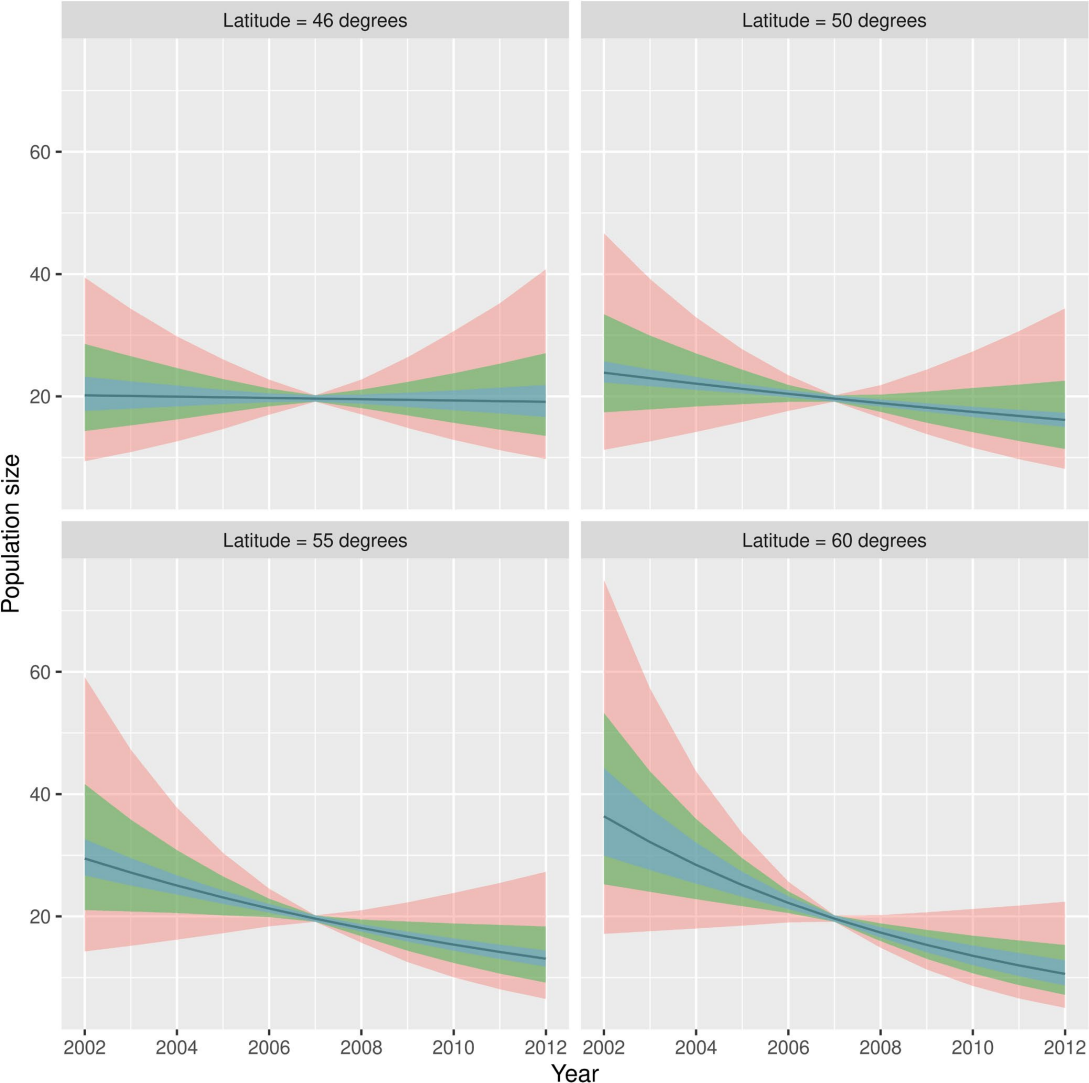
Expected trend in the number of common pochards counted in the northwestern flyway computed for average sites at 46°, 50°, 55°, and 60° latitude (curve and 95% confidence intervals in blue). The green area corresponds to the 95% prediction interval on the trends at the scale of the grid cell. The red area corresponds to the 95% prediction interval on the trends at the scale of the site. The blue area corresponds to the 95% credible interval on the mean trend. Note that all confidence and prediction intervals have minimal width in 2007 because the variable “year” has been centered to allow the fit of the model (i.e., *t* = 0 for year 2007, see *Material and Methods*)

Overall, the decline in northwestern pochard populations averaged 4.9% per year over the 11 years of the study period (SE = 0.6%), which represents a total decline of 42.3% (SE = 4%; Figure 3). Standard deviations of the random effects of slopes (Table 1) at the level of both the grid cell (*σ_b_*) and site (*σ_d_*) were quite large (Figure 2): for example, the typical yearly decline in a cell located at latitude 60° varied between $\left(1‐\exp\left(\beta_{0}+\gamma \cdot 60+\sigma_{b}\right)\right)=$ 8.8% and $\left(1‐\exp\left(\beta_{0}+\gamma \cdot 60‐\sigma_{b}\right)\right)=$ 14.6%. Similarly, the typical yearly decline in a sampling site located in a typical cell varied between 5% and 17.9% per year, reflecting strong differences in observed trends in numbers at these two sampling levels. Finally, the decline in numbers in a given site did not depend on the initial number of individuals counted at this site (the correlation coefficient between the intercept of the site and the slope of the relationship between year and number of individuals in this site was equal to 0; *R* = 0.0005 to be precise). In other words, all sites had the same probability to display any given decline independently of their initial state (in terms of number of wintering individuals).

## 4 DISCUSSION

In this study, we provide an example of the typical issues one may encounter when analyzing such large‐scale population censuses (here IWC) and provide a general framework to tackle them. In a preliminary step, we ran analyses that allowed not only detecting spatial autocorrelation but also helped characterizing its nature, that is, spatio‐temporal heterogeneity in sampling effort. In addition, these preliminary analyses showed that the decline in pochards observed during the study period did not depart from linearity. In a second step, we standardized the sampling units using a 75 × 75 km grid overlaid on the flyway of interest. This strategy proved efficient both to remove spatial autocorrelation (and therefore allowed estimating improved population trends regarding this potential source of bias) and to detect spatial (latitudinal) heterogeneity in the observed decline in the focal species in the area of interest.

Despite potential biases, one must recognize that without large‐scale and long‐lasting surveys like the International Waterbirds Census or its North American counterpart, the Mid‐Winter Survey, monitoring the conservation status of so many bird species would have simply been impossible (IUCN, 2020). Moreover, these monitoring schemes have proven crucial in the understanding of the impact of anthropogenic activities on wildlife (e.g., harvest and global warming) and the effect of mitigating actions (e.g., creation of protected areas and implementation of conservation action plans, Pavón‐Jordán et al., 2020; Marchowski et al., 2020). Unfortunately, because these surveys were initiated without proper sampling strategies and in a time (1935s) when data storage, digitizing, and statistical computing were still in their infancy, they may suffer from serious weaknesses that must be addressed properly, for example, using appropriate modeling framework, before any inference.

Bayesian hierarchical modeling has previously been used to assess bird population trends (including ducks) in North America based on MWS, CBC counts, and North American Breeding Bird Surveys (Link et al., 2006; Meehan et al., 2021; Sauer & Link, 2011; Soykan et al., 2016). However, the majority of these studies did not explicitly tackle spatial autocorrelation. Note that several authors also used a grid discretization of the study area to both standardize sampling units and model the spatial and temporal autocorrelation structure in the data (Bled et al., 2013; Meehan et al., 2019) into a Bayesian hierarchical model (conditional autoregressive and CAR structure). We did not model the autocorrelation using such models in our study because the standardization of sampling units through the grid approach proved fully efficient to remove spatial autocorrelation/structure. This does not mean, however, that a 75 × 75 km grid will prove efficient to remove spatial autocorrelation in all instances. Should they adopt our approach, future studies may have to adjust the size of grid cells and eventually resort to approach consisting to tackle spatial structure directly into the model (Meehan et al., 2019).

Thus, turning back to the focal species, it turns out that the annual decline in populations in the northwestern flyway averaged 4.9% ± 0.6 SE per year, a value similar to those computed previously with TRIM by Nagy et al. (2014) and BirdLife International (2015) (respectively, 5.9% per year on average over 2003–2012 and 4.4% per year over 2006–2015). Therefore, our more robust analysis confirms that pochard population experienced a sharp decline in the northwestern flyway.

Although our model proved to be efficient in circumventing many limits of the dataset, this modeling approach is not the panacea and cannot be considered as a replacement for a proper sampling of the population. Indeed, if we had the means to design a proper sampling design over the continental scale, we would also probably have defined another aim to the study. More precisely, we would have designed the sampling design and protocol to allow the estimation of the trend of the whole population size over the migration corridor. Due to the lack of common sampling strategy, we could not reach such an aim, and we designed a model allowing to estimate a spatial mean of the local trends over the corridor, which we used as a proxy of the pochard population trend. However, a spatial mean of the local trends is not exactly the same thing as the trend of the whole population size. Indeed, most of the pochard population is probably located in a restricted number of grid cells (e.g., in the Netherlands, where wetlands are numerous), whereas this spatial mean gives the same weight to all the grid cells of the corridor including those with a tiny fraction of the population. The local trends estimated in scarcely populated grid cells thus weigh too much in our global assessment. Our estimated population trend is therefore probably biased, although the population decline observed in most of the corridor gives us confidence in our qualitative assessment of the population status.

Numerous waterbird populations have been found (or are expected) to display range shifts toward the north/northeast owing to migration short stopping (reduction in migration distances) in response to rising temperatures (Elmberg et al., 2014; Visser et al., 2009). In these species, the numbers of wintering individuals usually experience declines in southwestern parts of the wintering range and increases in the northwestern parts, in Europe (Lehikoinen et al., 2013; Pavón‐Jordán et al., 2020). Our analyses do not support this view for pochards in the northwestern flyway where the decline in wintering individuals was in fact significantly stronger at northern than southern latitudes (−12% per year at 60° of latitude against stability at 46°). According to ringing recoveries, large proportions of pochards wintering in Northwestern Europe originate from beyond or up to the Ural Mountains (Folliot et al., 2020), casting serious doubt about the pertinence of the three putative flyways as delineated by Scott and Rose (1996). Thus, we cannot totally rule out that the decline in pochard in Northwestern Europe is due to individuals staying closer to their breeding grounds thereby evading the survey by remaining outside the putative flyway where most IWC counts are carried out. Moreover, it is also possible that increasing numbers of individuals are still spending the wintering season within the limits of their putative flyway, although closer to their breeding ground on (still) not surveyed sites particularly in the European part of Russia.

Previous studies addressing the problem of short stopping in waterbirds (e.g., ducks and wading birds) either computed geographic range shifts (Pavón‐Jordán et al., 2020) or based their reasoning upon crude comparisons between country in geographical changes in populations trends (Lehikoinen et al., 2013; Marchowski et al., 2020). Here, we provide a formal, reproducible framework for estimating how population trends vary over space showing that the observed decline is gradually increasing with latitude, which is similar in spirit to previous work on similar issues. We just changed the target of the inference, shifting from the estimation of a trend based on an estimate of total relative abundance on an area (Bled et al., 2013; Sauer & Link, 2011) to the estimation of an average of trends of local relative abundance because of the impossibility for us to suppose a spatial autocorrelation in relative abundance in sampled sites for waterbirds (i.e., no reason to suppose that the abundance will be similar in two close water points with different sizes). From a conservation and management point of view, therefore, our approach provides a convenient and flexible way for accurately assessing the status of species across space and time and eventually seeking its ultimate causes.

One of the most important finding of our study, which has important biological and statistical consequences, concerns the fact that population trends varied randomly across sites, and is not correlated with the intercept of our model, that is, with the average number of animals detected in the site. The rate of decline, therefore, did not vary according to mean numbers of individuals detected in the site. This is an interesting finding because if we suppose that the number of detected animals reflects the actual number of animals present in a site, then mean population trends can be assessed – in average – even in sites where few animals were detected, although this would still require a large number of such sites due to the large variability in the estimated trends from one site to another. This also gives us insights into the understanding of pochard's social processes during winter, and more particularly that large aggregations are not systematically favored by the birds as is often suspected. However, we concur that our approach cannot finely assess behavioral and/or social processes and that further research is needed to assess the underlying (social or behavioral) mechanisms of group structure and size.

## 5 CONCLUSIONS

The approach presented here may provide a convenient framework for quantitatively assessing spatial and temporal changes in population trends. However, although a significant improvement, our approach will probably never achieve the same performances (in terms of accuracy and precision) as a standardized sampling scheme. The take‐home message therefore is that census schemes such as the IWC should as far as possible aim at implementing a standardized sampling strategy allowing tackling the problems of sample representativeness and spatial heterogeneity of sampling effort before data collection. Only such a strategy would provide reliable estimates of both population sizes and trends. This issue was raised a few years ago with the Midwinter Inventory in the USA (Eggeman & Johnson, 1989; Heusmann, 1999). These surveys were abandoned in most states in favor of inventory with a standardized sampling protocol (see, e.g., Herbert et al., 2018; Masto et al., 2020; Whitaker et al., 2019).

## CONFLICT OF INTEREST

The authors have no competing interests.

## AUTHOR CONTRIBUTIONS

**Benjamin Folliot:** Conceptualization (equal); Data curation (equal); Formal analysis (equal); Writing – original draft (lead). **Alain Caizergues:** Conceptualization (equal); Supervision (equal); Writing – original draft (equal). **Adrien Tableau:** Conceptualization (equal); Data curation (equal); Writing – original draft (equal). **Guillaume Souchay:** Conceptualization (equal); Writing – original draft (equal). **Matthieu Guillemain:** Conceptualization (equal); Supervision (equal); Writing – original draft (equal). **Jocelyn Champagnon:** Conceptualization (equal); Supervision (equal); Writing – original draft (equal). **Clément Calenge:** Conceptualization (equal); Formal analysis (equal); Software (lead); Writing – original draft (equal).

### OPEN RESEARCH BADGES

This article has been awarded <Open Materials, Open Data, Preregistered Research Designs> Badges. All materials and data are publicly accessible via the Open Science Framework at https://doi.org/10.5281/zenodo.5710550; https://github.com/ClementCalenge/pochardTrend/commits/v1.

## Data Availability

We included all the code and data used in the R package pochardTrend (Digital Object Identifier: https://zenodo.org/record/5710550#.YkGTkOdBxhF) on GitHub at the following URL: https://github.com/ClementCalenge/pochardTrend. The reader can install this package in R using the function devtools::install_github ("ClementCalenge/pochardTrend", ref="main"). The pochardTrend package includes a vignette describing how the user can easily reproduce the model fit (available with the command vignette ("pochardTrend"), once the package has been installed).
